# Porous Fe_3_O_4_@BC Coupled with an Electric Field Facilitates Nitrogen Retention During Composting

**DOI:** 10.3390/nano16110689

**Published:** 2026-06-01

**Authors:** Meng Song, Keqing Li, Zhiqiang Yang, Siqi Zhang

**Affiliations:** 1 School of Resources and Safety Engineering, University of Science and Technology Beijing, Beijing 100083, China; msong@canre.org.cn; 2 Key Laboratory of Carrying Capacity Assessment for Resource and Environment, Ministry of Natural Resources, Beijing 100830, China; 3 School of Materials Science and Engineering, Tongji University, Shanghai 201804, China; zhqyang@tongji.edu.cn

**Keywords:** organic fertilizer, nitrogen conversion, emission reduction, microorganisms, NH_3_ emissions

## Abstract

This study synthesized a novel Fe_3_O_4_/biochar composite (Fe_3_O_4_@BC) characterized by a porous structure and a high electron-donating capacity. The effect of Fe_3_O_4_@BC on ammonia emission and nitrogen loss during electric-field-assisted composting was investigated, and its underlying mechanism in nitrogen transformation was elucidated. Results demonstrated that the addition of an appropriate amount of Fe_3_O_4_@BC reduced cumulative NH_3_ emission and total nitrogen loss by 30.00% and 4.03%, respectively. The favorable changes in gas emissions could be attributed to Fe_3_O_4_@BC-mediated modulation of key core microbial taxa. Under the electric-field-coupled condition, Fe_3_O_4_@BC addition significantly promoted the proliferation of *Actinobacteria*, such as *Thermobifida* and *Corynebacterium*, during the high-temperature phase, while concurrently suppressing the activity of *Firmicutes*. The shift in core microbial communities optimized key nitrogen transformation processes, including ammonification and nitrification, ultimately leading to reduced NH_3_ emission. This study highlights the application potential of Fe_3_O_4_@BC in enhancing nitrogen retention and mitigating emissions during composting.

## 1. Introduction

Electric field-assisted composting can be regarded as a novel and efficient method for biological waste treatment and resource recovery. This technology has been proven to accelerate the fermentation process of agricultural wastes and improve composting efficiency [1]. However, this process is frequently linked to substantial nitrogen loss, unpleasant odour, and greenhouse gas emissions [2], particularly NH_3_ and N_2_O. Furthermore, these factors have the capacity to diminish the nutrient content of compost products, thereby compromising their practical utility [3]. The effective control of nitrogen loss in electric field-assisted composting technology represents a significant challenge. The predominant mechanism of nitrogen loss during the composting process is the cumulative volatilisation of NH_3_, which predominantly occurs during the high-temperature stages when microorganisms are highly active [4]. Another significant nitrogen loss pathway that has been the focus of research is the emission of N_2_O.

The utilisation of exogenous additives to mitigate nitrogen loss during electric-field-assisted composting is widely recognised as the most effective approach [5]. Biochar (BC) possesses abundant cyclic structures at the molecular level and contains a large number of surface functional groups, including quinone, hydroquinone and anthraquinone groups, making it a potential electric field-composting friendly material [6]. These functional moieties serve as effective electron shuttles, facilitating rapid electron transfer during organic matter biodegradation. According to Sanchez-Monedero et al., supplementing composting systems with 10% oak-derived biochar can reduce nitrogen loss by up to 86.4% [7]. Such mitigation is intimately associated with the protective effect of biochar on nitrogen-containing compounds, suppressed denitrification, and enhanced nitrification during composting. Specifically, under an electric field environment, the addition of electric field-favorable materials has been proven to promote the proliferation of electroactive bacteria (EABs). In terms of its effects on nitrogen transformation, certain specific microorganisms rapidly reproduce and achieve selective enrichment by attaching to the physicochemical structures on the surface of electrochemical materials [8]. Meanwhile, the activities of dominant nitrifying and denitrifying bacteria are inhibited, thereby significantly reducing N_2_O emissions [8]. Nevertheless, the influence of biochar addition on NH_3_ volatilization during electric field composting remains rarely investigated.

In comparison with conventional additives, mineral additives, such as manganese dioxide, ferric oxide, and magnesium oxide, have garnered mounting attention on account of their substantial advantages [9,10]. Ferric oxide, when utilised as a functional additive, provides essential iron (Fe) for microorganisms, while concurrently exhibiting excellent redox ability [11,12]. This ability promotes the growth of Fe-reducing bacteria and facilitates electron transfer from the cell exterior to other microorganisms [13]. As posited by Zhang et al., magnetite has been demonstrated to exert a substantial effect on the augmentation of microbial activity during the composting process [14]. Yang et al. demonstrated that the addition of magnetite resulted in a decrease in NH_3_ emissions during composting, whilst concurrently increasing N_2_O emissions [15]. Consequently, the reduction of N_2_O emissions with ferric oxide remains an issue that requires resolution. In overcoming this limitation, the incorporation of biochar conditioners has been considered because it may be more effective in reducing N_2_O emissions during electric field-assisted composting [16].

The hypothesis of this study is that, during composting, the combination of iron-based porous materials (pyrolyzed iron-based porous material, Fe_3_O_4_ with biochar) will generate a synergistic enhancement effect by integrating both adsorptive and conductive properties. Biochar has been demonstrated to function as an electron shuttle, thereby promoting the growth of electroactive bacteria (EABs) and suppressing nitrification–denitrification. This process has been shown to result in a reduction in nitrous oxide emissions. Iron oxides have been shown to reduce NH_4_^+^ levels in the compost, thereby decreasing NH_3_ emissions [14]. Compared with existing studies, the integration of iron oxide and biochar shows greater potential for simultaneously mitigating NH_3_ and N_2_O emissions during electric field-assisted composting. However, the application of ferric oxide and biochar in aerobic fermentation under electric field composting is still uncommon, and its underlying mechanisms remain largely unexplored.

This study aimed to optimize aerobic composting by employing a composite material synthesized from Fe_3_O_4_ and BC, so as to synchronously mitigate emissions of NH_3_ and N_2_O. Meanwhile, the effects of Fe_3_O_4_@BC on nitrogen transformation and key microbial communities during composting were systematically investigated. The results indicated that the addition of Fe_3_O_4_@BC altered the microbial community structure at both the phylum and genus levels, and further regulated key nitrogen transformation processes such as ammonification.

## 2. Materials and Methods

### 2.1. Synthesis of Fe_3_O_4_@BC

Fe_3_O_4_@BC was synthesized following the protocol reported by Siew et al. [17]. An aqueous solution containing trimesic acid (C_9_H_6_O_6_, 0.03 M, Macklin, Shanghai, China) and caustic soda (NaOH, 0.09 M, Macklin, Shanghai, China) was added dropwise into the Fe(NO_3_)_3_·9H_2_O solution (0.03 M, Macklin, Shanghai, China) in a volumetric proportion of 3:1. At room temperature, the mixture was stirred for 1 h to form the reaction substrate. The obtained suspension was repeatedly centrifuged and filtered using a constant-speed magnetic stirrer (85-2A, Jiangsu Shuxing, Shaoxing, China), qualitative filter paper (pore size: 100 μm), and a refrigerated centrifuge (H2050R, Xiangyi, Shanghai, China) to collect solid products. The solids were dried in a vacuum oven (FZG-20, Nanjing Hewang Machinery Equipment Technology, Nanjing, China) at 80 °C for 24 h to obtain iron-based gel material (C_9_H_3_FeO_6_). Subsequently, the C_9_H_3_FeO_6_ was thermally treated at 500 °C maintained in air for 0.5 h to form porous iron oxide. The obtained iron oxide product was mixed with coconut shell particles sourced from Hainan, China. The mixture was subjected to carbonization treatment at 550 °C for 120 min in a nitrogen atmosphere, with a programmed heating rate of 2 °C per minute. The porous Fe_3_O_4_@BC composite was thus successfully prepared.

### 2.2. Composting Procedure

In the present research, three sets of consistent composting reaction devices were utilized for experimental tests. Each reactor consisted of a steel cylindrical vessel (with a diameter of 0.45 m, a height of 0.45 m and a working volume of 60 L), with its inner surface coated with conductive adhesive tape. A graphite rod (40 cm length, 1.5 cm radius) serving as the cathode was fixed at the center of each reactor, while conductive tape was used as the anode. The main experimental device reduces heat loss by wrapping sound insulation cotton. A circumferential electric field was supplied at a constant direct current (DC) voltage of 2 Volts. Continuous forced aeration was applied throughout the experimental process at an aeration of 500 mL·kg^−1^·min^−1^.

A 27-day aerobic composting trial was carried out using pig manure as the primary substrate and straw as a bulking agent. Fresh pig manure was obtained from a local swine farm in Beijing, China, while dried corn straw was collected from farms in Jiangsu Province. The two feedstocks were mixed at a dry–weight ratio of 4:1 to adjust the initial moisture content to 60% and the C/N ratio to 25. Three treatments were established: a control group without Fe_3_O_4_@BC (CK); a treatment amended with 1 wt% Fe_3_O_4_@BC (dry weight basis, T1); and a treatment amended with 3% Fe_3_O_4_@BC (wet weight basis, T2). All treatments were performed in 60-L lab-scale composting reactors designed and constructed by our research group.

### 2.3. Characterization of Fe_3_O_4_@BC

The morphology of the materials was examined using a scanning electron microscope (SEM, InTouchScope, Tokyo, Japan) [18]. X-ray diffraction characterization was performed on a Bruker’s diffractometer (D8 Advance, Bruker, Ettlingen, Germany) to acquire the crystalline structure patterns of the samples [19]. Fourier transform infrared spectroscopy (FTIR) was adopted using a Nicolet Summit spectrometer (Nicolet iS50, Thermo Fisher, Waltham, MA, USA). The specific surface area (SSA) and pore structure parameters of Fe_3_O_4_@BC were determined by a surface area and porosity analyzer (ASAP 2460, Micromeritics, Shanghai, China) [18]. Following the analytical method of He et al., the electron-donating capacity (EDC) of the materials was determined via chemical titration [18].

### 2.4. Physicochemical and Microbial Community Analysis

Solid samples of around 300 g were collected at days 0, 3, 7, 11, 15, 21 and 27 throughout the experiment via the five-point sampling technique. During composting, the temperature of the compost was measured at predetermined times daily. The electrical conductivity (EC) and pH of the reactor body were measured by conductivity meter (Leici DDS-307A, Shanghai, China) and pH detector (Mettler Toledo Fe20, Shanghai, China). Oxygen (O_2_) concentration at the exhaust of the composting reactor was monitored using a portable electrochemical gas analyzer (Biogas 5000, Geotech, Warwickshire, UK). The seed germination index (GI) was calculated following the procedure reported by Wang et al. [19].

The contents of various forms of nitrogen in compost were determined according to the method described by Liao et al. [20]. For organic nitrogen determination, samples were dried at 120 °C for 24 h, ground, and then measured by the Kjeldahl method [21]. Fresh compost samples were extracted with 2 mol/L potassium chloride solution, and the concentration of inorganic nitrogen in compost samples was determined by flow Auto-analyzer (Tech-II, SEAL, Norderstedt, Germany). NO_2_ concentration was collected according to the alternate schedule [22]. It was monitored every day in the first week, and then every two days. Chromatographic detection technology (6890 n, Agilent, Santa Clara, CA, USA) was used for analysis [22].

In order to quantify the migration pathways and loss routes of N during the composting process, the mass balance method was used to calculate the nitrogen changes in the Fe_3_O_4_@BC treatments [23]. The Kjeldahl method was used to determine the variations in organic nitrogen at the initial phase (Day 0) and terminal phase (Day 27), which was defined as the nitrogen involved in transformation. The concentrations of inorganic nitrogen including NH_4_^+^, NO_2_^−^ and NO_3_^−^ were recorded as process data, and their contents on Day 27 were regarded as the final inorganic nitrogen (Inorganic-N). The emissions of NH_3_ and N_2_O were considered gaseous nitrogen loss, and the residual difference in total nitrogen throughout the composting process was calculated as other nitrogen loss (Other loss).

### 2.5. Microbial Community Analysis

Microbial detection and analysis were performed based on well-established experimental methods, and the detailed procedures are provided in the Supplementary Materials [23]. Target DNA was extracted from samples using Fast SPIN DNA extraction kit (Omega Bio-tek, Norcross, Georgia, Norcross, GA, USA), qualified by agarose gel electrophoresis and NanoDrop2000 spectrophotometer (Thermo Scientific, Waltham, MA, USA) [24]. The V3-V4 region of 16S rRNA gene was amplified via PCR with primers 338F (5′-ActCCtacGGgaggCAGCAGG-3′) and 806R (5′-GGACTACHVGGGTWTCTAAT-3′) [25]. Purified PCR products were constructed into libraries and sequenced on the Illumina MiSeq PE300 platform (Majorbio, Shanghai, China) adopting NEXTFLEX nova-5149 kit. Raw sequencing data were subjected to quality control with fastp (V0.20.1) software [26]. Processed sequences were clustered into operational taxonomic units (OTUs) using UPARSE (V7.1) software. Species annotation was conducted at a similarity threshold of 70%, and community composition of each sample was analyzed at various taxonomic levels. The Functional Annotation of Prokaryotic Taxa (FAPROTAX, V1.2.10) database was adopted to annotate nitrogen metabolism functions of microbial communities, and the species abundance table obtained from 16S rRNA sequencing was converted into functional abundance table [27].

### 2.6. Statistical Analysis

Mathematical analysis of compost and relevant experimental data was completed using SPSS 16.0 software. All data were visualized and analyzed via Office 2017, Origin 2022, and the Majorbio platform (https://www.majorbio.com).

## 3. Results and Discussion

### 3.1. Material Characterization

**Figure 1:**
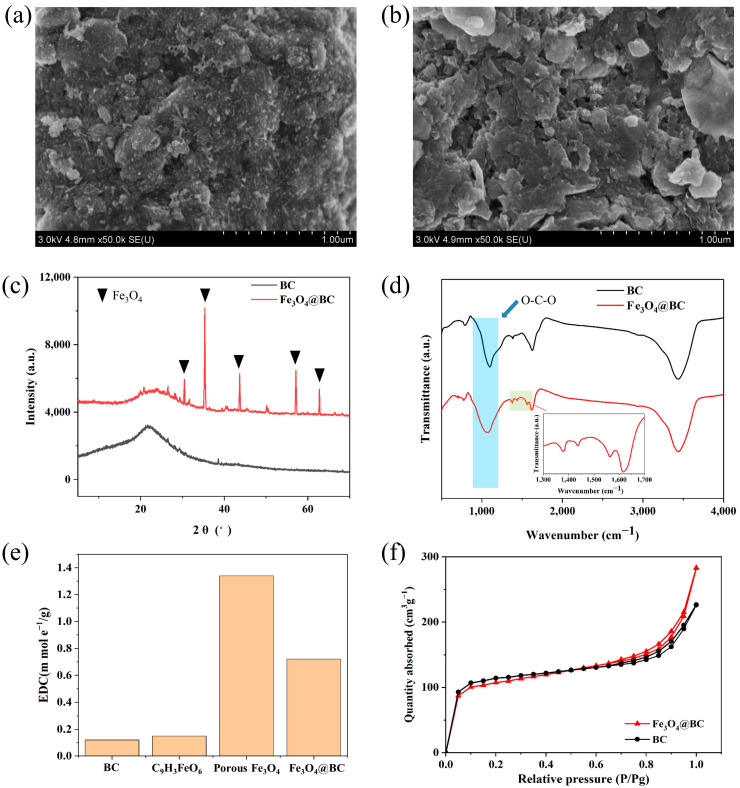
SEM images of BC (**a**), Fe_3_O_4_@BC (**b**); the XRD pattern (**c**) and FTIR (**d**); EDC of BC, Fe-BTC, porous Fe_3_O_4_, and Fe_3_O_4_@BC (**e**); nitrogen adsorption–desorption isotherms of BC and Fe_3_O_4_@BC (**f**). In general, compared with pure Fe_3_O_4_ and pristine biochar [7], Fe_3_O_4_@BC exhibits a larger specific surface area and richer surface functional groups, which facilitates the adsorption and reduction of ammonia emissions and provides additional active sites for microbial reactions during composting. Meanwhile, the EDC value of Fe_3_O_4_@BC is much higher than that of unmodified BC, which is beneficial to the composting process and microbial interspecific electron transfer under electric field conditions.

The morphological characteristics of raw BC and the synthesized Fe_3_O_4_@BC composite were systematically characterized via SEM, whereby the pristine BC sample was observed to possess a unique layered and wrinkled structural configuration while the modified Fe_3_O_4_@BC material exhibited uniform dispersion of Fe_3_O_4_ particles across the entire biochar surface (Figure 1a,b). The crystal structure features of BC and Fe_3_O_4_@BC were further validated through XRD analysis, and the resulting diffraction spectra revealed six prominent characteristic peaks of Fe_3_O_4_@BC at 2θ angles of 30.4°, 35.3°, 43.6°, 53.3°, 57.1°, and 62.7°, all of which were highly consistent with the standard crystalline diffraction signals of ferroferric oxide reported in previous studies [28]. The combination of consistent morphological observations and standard XRD characteristic diffraction peaks comprehensively demonstrates the successful fabrication and structural integrity of the Fe_3_O_4_@BC composite material in this study.

FTIR analysis was performed to identify the specific functional groups on the surfaces of BC and Fe_3_O_4_@BC. As shown in Figure 1d, distinct absorption bands associated with the asymmetric stretching vibration of O-C-O were detected near 1050 cm for both BC and Fe_3_O_4_@BC. Compared with pristine BC, several weak absorption peaks emerged for Fe_3_O_4_@BC around 1500 cm, which could be attributed to the metal carbonyl complexes (M-CO) induced by Fe_3_O_4_ modification [29]. In summary, porous Fe_3_O_4_ significantly optimized the types and abundance of functional groups on the biochar surface. For composting applications, the modified material can provide additional active sites for microorganisms.

EDC serves as a critical index for assessing the electrochemical performance of various materials. Numerous studies have demonstrated a significant correlation between fermentative microbial activity, nitrogen transformation pathways, and the EDC of composting amendments [18]. As shown in Figure 1e, the EDC values for BC, Fe-BTC, porous Fe_3_O_4_, and Fe_3_O_4_@BC are 0.15, 0.13, 1.54, and 0.72 m mol e^−^ g^−1^, respectively. XRD analysis revealed that the formation of iron oxide as the dominant phase in Fe_3_O_4_ after the pyrolysis of Fe-BTC substantially enhances its electron-donating ability [30]. The EDC of Fe_3_O_4_@BC is 480% of that of BC, a result closely linked to the doping with Fe_3_O_4_. Compared to BC, Fe_3_O_4_@BC facilitates electron transfer during composting.

The SSA and pore structure of composting amendments significantly influence ammonia emissions, as they govern the physical adsorption capacity of gases and the transport capability of electrons and ions within the compost matrix. Table 1 presents the pore texture parameters of BC and Fe_3_O_4_@BC. The SSA of Fe_3_O_4_@BC is 14.35% greater than that of BC. The adsorption–desorption isotherms of the as-prepared materials presented in Figure 1f reveal that all tested samples possess favorable adsorption performance under low relative pressure conditions, and the modified Fe_3_O_4_@BC composite exhibits consistently higher adsorption capacity than pristine BC throughout the whole adsorption process, which is indicative of improved pore structure development and increased SSA after Fe_3_O_4_ modification. In the low-pressure region (*p*/*p*_0_ < 0.1), both materials exhibit a distinct slope, indicating the presence of micropores that provide additional adsorption sites. At higher relative pressures (0.45–0.85), the slope of the adsorption curve for Fe_3_O_4_@BC (classified as an H4-type hysteresis loop) exceeds that of BC, accompanied by a pronounced hysteresis loop. This indicates that Fe_3_O_4_@BC possesses a more developed mesoporous network. Such mesoporous structures provide favorable channels for charge transmission, which is beneficial to optimizing the electrochemical properties of the prepared material [31].

### 3.2. Variation in Physicochemical Indices During Composting

**Figure 2:**
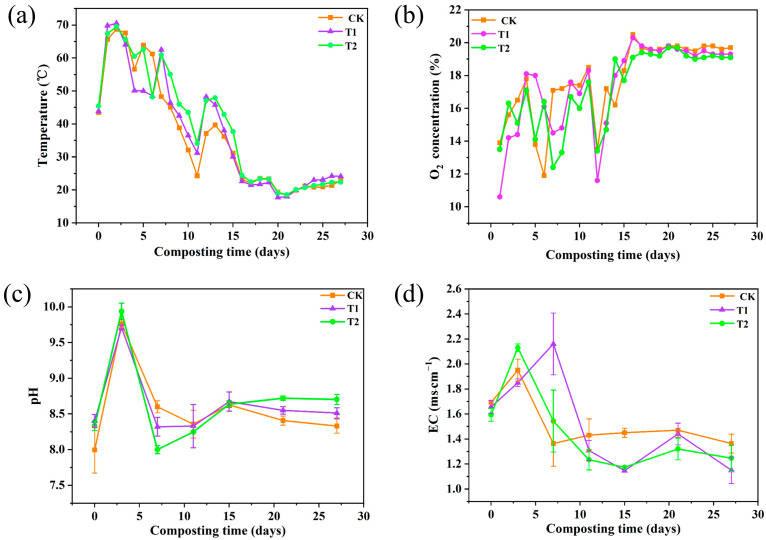
Changes in physical and chemical properties of compost (**a**) temperature, (**b**) oxygen, (**c**) pH, and (**d**) EC.

Changes in composting temperature can directly reflect the biochemical process of materials and microbial activity during composting, and Figure 2a shows that the pile temperature of all groups increased rapidly with the progress of composting, exceeded 55 °C after the second day, remained stable for 7 to 8 days, and reached a peak value of approximately 70 °C, a temperature duration that is sufficient to eliminate harmful microorganisms and complies with standard composting regulations; oxygen content, which served as another key monitoring indicator in this experiment, fluctuated throughout the composting process with a trend of initial decline followed by two successive increases (Figure 2b), a phenomenon that can be explained by dynamic variations in oxygen consumption driven by shifts in microbial communities, wherein the rapid proliferation of microorganisms after oxygen supplementation into the reactor significantly elevated oxygen consumption and thus reduced the oxygen content in exhaust gas, while the intensified degradation of compost materials and intensified microbial competition subsequently decreased microbial oxygen utilization efficiency and gradually raised the exhaust oxygen content. As illustrated in Figure 2c, the fluctuation of pH values during composting was closely correlated with organic matter stability and nitrogen loss, and all three treatment groups exhibited consistent pH variation trends characterized by a transient increase during the thermophilic phase, a gradual decrease in the subsequent period, and final stabilization at a relatively uniform level, with a prominent sharp pH elevation observed in all groups during the thermophilic phase due to the substantial transformation of organic nitrogen into inorganic nitrogen in compost materials. EC values, which objectively reflect the inorganic salt content of compost piles, can indicate the maturity degree and biological toxicity level of organic fertilizers, and the experimental results showed that the treatment groups presented slightly higher dynamic EC values than the control group during composting with negligible differences in final EC values, which were recorded as 1.36 ms/cm for the control group, 1.15 ms/cm for the T1 group, and 1.24 ms/cm for the T2 group, all of which met the standards for pollution-free composting [32]. Overall, the addition of Fe_3_O_4_@BC exerted no negative effects on the composting process, and all physicochemical indicators of composting satisfied the maturity requirements, demonstrating that Fe_3_O_4_@BC supplementation cannot significantly alter the conventional physicochemical properties of compost piles and that its potential regulatory effects on nitrogen transformation during composting are not mediated by intuitive physicochemical pathways such as pH and oxygen content variation [33].

### 3.3. Variations in NH_4_^+^-N and NO_3_^−^-N Contents

**Figure 3:**
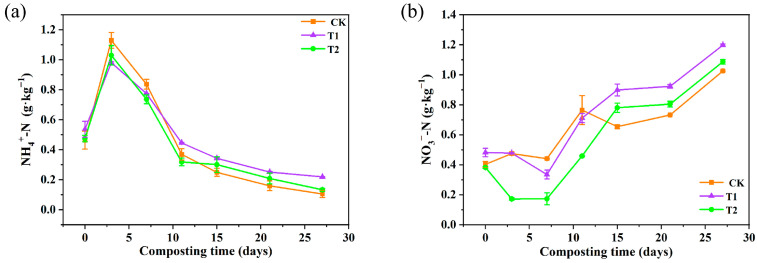
Changes of NH_4_^+^-N (**a**) and NO_3_^−^-N (**b**) during composting.

Inorganic nitrogen exists in various forms during composting, among which ammonium nitrogen (NH_4_^+^-N) serves as the predominant component and acts as a core substance governing nitrogen transformation processes including organic nitrogen mineralization and ammonia volatilization [20]. The dynamic variations in NH_4_^+^-N content throughout composting are presented in Figure 3, where all three groups exhibited a consistent trend of initial increase followed by progressive reduction, with the NH_4_^+^-N concentrations of all groups peaking on day 3 at 1.13 g·kg^−1^ in the CK group, which was 15.3% and 9.7% higher than those in the T1 and T2 groups, respectively; the NH_4_^+^-N content of all treatments gradually declined thereafter and finally stabilized at relatively low levels in the late composting stage, with final values of 0.11 g·kg^−1^, 0.22 g·kg^−1^, and 0.13 g·kg^−1^ for the CK, T1, and T2 groups, correspondingly. Notably, the NH_4_^+^-N concentration in the T1 group slightly increased after day 7 and exceeded those in the CK and T2 groups, though the overall nitrogen content at this stage remained markedly lower than that during the high-temperature period. Combined with previous research findings, the appropriate addition of Fe_3_O_4_@BC appears to weaken the mineralization of nitrogen-containing organic matter during the high-temperature stage while strengthening this process in the late composting stage, which can originate from the microbial regulation effect induced by Fe_3_O_4_@BC during composting.

During the early composting phase, unfavorable physicochemical conditions and insufficient available energy sources greatly suppressed the activity of nitrifying bacteria. For this reason, the nitrate nitrogen (NO_3_^−^-N) concentration in all compost piles remained at a low and steady level without obvious fluctuations in the first seven days. As composting proceeded, the gradual decline in compost temperature and pH facilitated the succession of microbial communities, where nitrifying bacteria gradually replaced ammonia-oxidizing bacteria and became the dominant microorganisms. This microbial shift effectively promoted the rapid accumulation of NO_3_^−^-N content in all experimental groups during the middle and late composting periods. The CK, T1 and T2 groups were 1.03, 1.20, and 1.09 g·kg^−1^. As a crucial nutrient component of organic fertilizers, NO_3_^−^-N represents one of the core concerns in this study, and the experimental results revealed that although the NO_3_^−^-N contents of the T1 and T2 groups remained at low levels in the early composting stage, they increased substantially in the subsequent stage and eventually surpassed that of the CK group, indicating that the supplementation of Fe_3_O_4_@BC exerts a certain regulatory effect on the dynamic variation of NO_3_^−^-N during composting.

### 3.4. Release of NH_3_ and N_2_O

**Figure 4:**
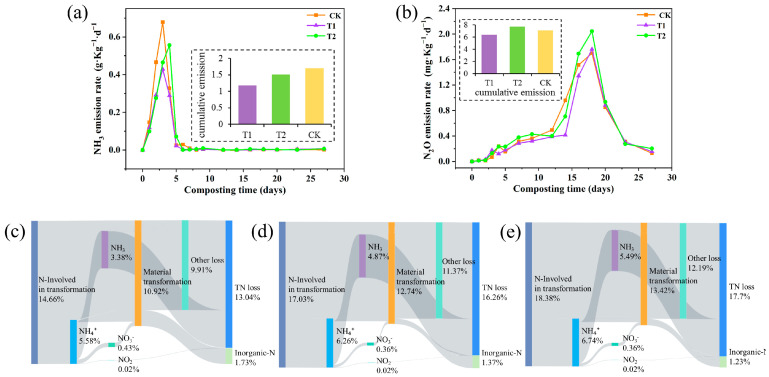
Variations in NH_3_ emission rate and its cumulative emissions (**a**), N_2_O emission rate and its cumulative emissions (**b**); loss pathways of nitrogen in T1 (**c**), T2 (**d**), and CK (**e**).

Ammonia volatilization is a major cause of nitrogen loss during composting [6]. The dynamic NH_3_ emission characteristics of each group are shown in Figure 4a. Both CK and T1 reached their peak ammonia volatilization rates on day 3, while T2 peaked on day 4. The peak values of CK, T1, and T2 were 0.68 g·kg^−1^·d^−1^, 0.43 g·kg^−1^·d^−1^, and 0.56 g·kg^−1^·d^−1^, respectively. Compared with CK, the T1 group showed a significant decrease of 36.76% in peak ammonia emission, while T2 exhibited no obvious change. The T2 group contained a higher dosage of Fe_3_O_4_ with excellent electron transmission and adsorption capacity than T1. The slightly higher NH_3_ volatilization in T2 was possibly due to the enhanced catalytic effect of excessive Fe_3_O_4_@BC [34]. This catalytic effect accelerated the degradation of nitrogen-containing organic matter and promoted ammonia release. In terms of cumulative NH_3_ emission, the T1 and T2 groups recorded 1.19 g·kg^−1^· and 1.51 g·kg^−1^, which were lower than the 1.70 g·kg^−1^ of the CK group. The T1 group with 1% Fe_3_O_4_@BC showed the lowest cumulative ammonia emission, with a 30% reduction compared with CK and T2. These results indicated that 1% Fe_3_O_4_@BC addition effectively reduced NH_3_ volatilization and facilitated nitrogen sequestration. Combined with the previous finding that Fe_3_O_4_@BC hardly changes conventional compost physicochemical indexes, its regulation on nitrogen transformation in electric field-assisted composting may depend mainly on microbial modulation rather than physicochemical effects. Further research is required to clarify the mechanism of Fe_3_O_4_@BC on microbial community structure in electric field composting systems.

As illustrated in Figure 4b, all three experimental groups exhibited consistent variation trends in N_2_O emissions, with low emission levels observed at the initial stage and thermophilic phase of composting [35]. The generation of N_2_O is closely associated with nitrification and denitrification processes. Although anaerobic environments suitable for the activities of nitrifiers and denitrifiers were formed during certain composting periods, excessively high temperatures inhibited the proliferation and metabolic activity of these functional microorganisms, thereby restricting the generation and emission of N_2_O throughout the composting process [36]. With the progression of composting, the temperature and pH of compost piles gradually decreased, and nitrifying bacteria gradually replaced ammoxidation bacteria to become the dominant microbial community. This shift facilitated the transformation of ammonium nitrogen into nitrate nitrogen, accompanied by a significant rise in N_2_O concentration across all treatment groups. According to the statistical results in the inset of Figure 4b, the cumulative N_2_O emissions of CK and the other two groups were 7.14 mg, 6.38 mg and 7.70 mg. The incorporation of 1% Fe_3_O_4_@BC resulted in a reduction in cumulative N_2_O emissions, which was likely attributable to the adsorption capacity of Fe_3_O_4_@BC (Figure 1e,f). Furthermore, the loose and porous structure of modified biochar can promote microbial activity and related enzymatic reactions, which is consistent with the regulatory effect of zeolites during pig manure aerobic composting [37]. Iron minerals loaded on biochar can effectively improve the redox reaction activity of compost systems [38]. The surface Fe on modified biochar can interact with NO_3_^−^ and NO_2_^−^, thereby promoting the terminal denitrification reaction that generates N_2_ [39]. Accordingly, the loaded iron species on biochar likely promoted the reduction of NO_3_^−^ to N_2_ This biochemical process is responsible for the reduced N_2_O emissions in the late stage, as supported by the synchronous decline in NO_3_^−^-N content.

The mass balance calculation results (Figure 4c,e) illustrate the fate and loss pathways of nitrogen in all treatment groups. In comparison with the control, the Fe_3_O_4_@BC treatment resulted in a reduction in total nitrogen loss, with the CK, T1 and T2, groups exhibiting respective reductions of 17.07%, 13.04% and 16.26%. The reduction in nitrogen loss by Fe_3_O_4_@BC was primarily achieved by decreasing NH_3_ volatilization. Moreover, Fe_3_O_4_@BC addition facilitated the nitrification process and drove the transformation of partial NH_4_^+^-N to NO_3_^−^-N. The final NO_3_^−^-N content of the T1 was 19.44% higher than that of the CK. The influence not only reduced NH_3_ volatilisation but also supplied a more readily absorbable form of nitrogen for plants, thereby enhancing nitrogen utilisation efficiency.

### 3.5. Biological Mechanism During Composting

#### 3.5.1. Microbial Community Structure

**Figure 5:**
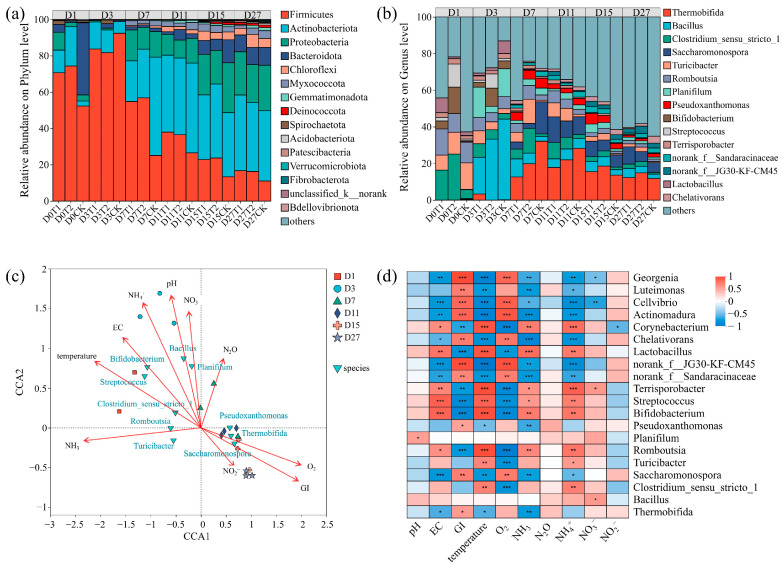
Changes in the relative abundances of bacterial phyla (**a**), gene (**b**); PCoA analysis of the bacterial communities based on the OTUs of 16S rRNA gene (**c**); Spearman Correlation Heatmap of three treatments during composting (**d**). Note: ***, **, * indicate significance at the 1‰, 1%, and 1% levels, respectively.

In the early stages of composting, the most prevalent bacterial species were identified as *Firmicutes*, *Actinobacteriota*, *Proteobacteria*, and *Bacteroidota* (Figure 5a). At the thermophilic stage, the relative abundances of *Actinobacteria* in D3T1 (14.86%) and D3T2 (12.40%) were significantly higher than that in D3TCK (6.5%). *Actinobacteria* serves as an essential microbial phylum involved in nitrogen transformation throughout composting, which greatly affects the structural composition of microbial communities [40]. Therefore, our finding that Fe_3_O_4_@BC addition affected *Actinobacteria* abundance suggests that this composite material has the potential to modulate the microbial composition in compost. *Firmicutes* exhibited a rapid decline at maturity, with the most significant decrease observed in the CK (10.97%) group, followed by the T2 (16.25%) and T1 (16.66%) groups. The relative abundance of *Actinobacteriota* increased markedly in experimental groups. The T1 possessed the highest relative abundance of *Actinobacteriota* at 41.72%, followed by the T2 group (37.90%) and CK group (38.78%). The relative abundance of *Bacteroidota* increased in all groups, with the CK group recording the highest value of 9.8%, followed by the T2 group (9.4%) and T1 group (9.2%). *Bacteroidota* are functionally associated with denitrification processes, which explains the higher nitrous oxide emissions observed in the CK [41]. In addition, the dominant proportion of *Chloroflexi*, a microbial phylum with potential nitrogen cycle functions, increased significantly during the compost maturation stage [42]. Specifically, the T2 group achieved the maximum *Chloroflexi* relative proportion of 7.11% on day 27.

Figure 5b illustrates the variations in dominant microorganisms at the genus level (relative abundance > 3%) across all experimental groups. The initial composting stage was predominantly colonized by *Clostridium-sensu-stricto*, *Turicibacter*, *Romboutsia*, and *Bifidobacterium*, all of which play essential roles in organic matter degradation. During the thermophilic stage, the relative abundance of *Bacillus* in D3T1 and D3T2 was significantly lower, being 33.24% and 19.94% lower than that in D3CK. The D3T2 treatment substantially enriched *Bifidobacterium*, while the relative abundance of this genus remained below 5% in other groups. The level of *Thermobifida* in D3T1 was found to be significantly higher than that observed in D3T2 and D3CK. Furthermore, the proliferation of *Corynebacterium* and *Thermobifida* was augmented by Fe_3_O_4_@BC during the elevated temperature phase. The results of this study suggest that Fe_3_O_4_@BC has the capacity to increase the relative proportion of certain bacterial groups involved in nitrogen transformation. At the maturation stage, the relative proportion of Bacillus in D27T1 (5.16%) was higher than that in D27T2 (3.6%) and D27CK (2.3%). Furthermore, the relative proportion of *Saccharomonospora* in the D27T1 (9.4%) treatment group was higher than that in the D27T2 (5.3%) and D27CK (5.3%) groups. Considering that *Bacillus* and *Saccharomonospora* are key participants in the ammonification reaction, this also explains the experimental trend of ammonia emissions in the T1 group, which were lower during the high-temperature stage and higher during the maturation stage (Figure 3a). The results of this study indicate that Fe_3_O_4_@BC can regulate the relative abundance of certain bacterial communities involved in nitrogen transformation, thereby affecting ammonia emissions during composting.

#### 3.5.2. Correlation Analysis of Factors Affecting Composting

A significant correlation existed between material physicochemical properties, microbial communities and gas emissions throughout the composting process (Figure 5c). A correlation analysis was conducted between temperature, oxygen content, NH_3_, N_2_O, and the physical and chemical properties of composting products EC, pH, NH_4_^+^, NO_3_^−^. During the composting process, a positive correlation was observed between NH_3_ levels and temperature, pH, and NH_4_^+^. This suggests that NH_3_ emissions increased significantly at elevated temperatures and pH levels. NH_3_ volatilisation exhibited a significant negative correlation with GI and NO_2_^−^, suggesting that NH_3_ emission was inversely related to compost maturity. As the quantity of biodegradable materials declined during the advanced phases of composting, the metabolic activity of the microorganisms diminished. Consequently, the oxygen utilization rate was found to be low.

The spearman correlation heatmap revealed a correlation between NH_3_ emissions and the presence of *Thermobifida*, *Saccharomonosporag*, and *Clostridium_sensu_stricto* (Figure 5d). The bacterial groups in question exhibited a proportional relationship with oxygen concentration, thus indicating that NH_3_ emissions are linked to oxygen levels and aerobic microorganisms. This observation is consistent with the previous description, in which the incorporation of Fe_3_O_4_@BC enhanced the porosity of the compost, improved oxygen utilisation, and acted as an electron shuttle to expedite the degradation of organic matter. The NO_3_^−^ variable demonstrated a positive correlation with *Bacillus*, which may have exerted an influence on N_2_O emissions.

### 3.6. Functional Prediction of Bacterial Community and Network Analysis

**Figure 6:**
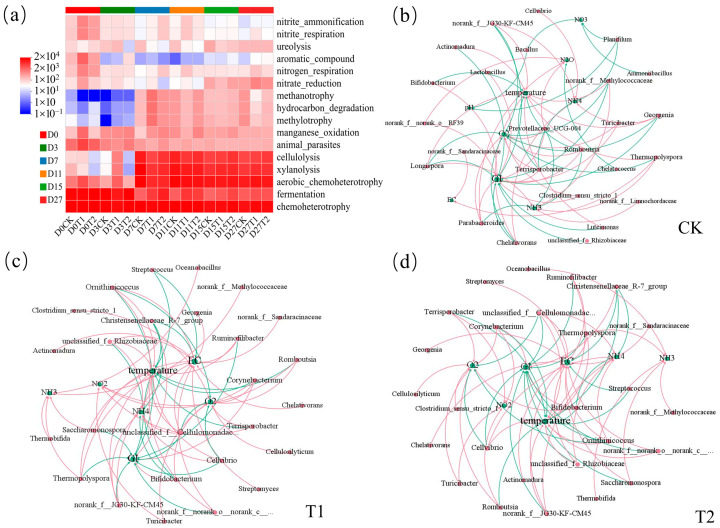
The FAPROTAX analysis of bacterial communities (**a**) and Network analysis of NH_3_ and N_2_O emission with environmental factors and microorganism of CK (**b**), T1 (**c**) and T2 (**d**).

The effect of Fe_3_O_4_@BC on nitrogen transfer during composting was investigated using FAPROTAX (Figure 6a). Low levels of nitrate, nitrite and nitrous oxide, as well as weak denitrification activity, were observed in the early composting stage. In the early composting phase, the contents of nitrate, nitrite and nitrous oxide, as well as the abundance of denitrification-related microorganisms, remained at relatively low levels. By contrast, functional activities of nitrite ammonification, nitrite respiration, nitrogen respiration, and nitrate reduction were highly active during this period. Moreover, the amendment of Fe_3_O_4_@BC effectively promoted nitrogen fixation capacity. Continuous aeration maintained consistent bacterial metabolic activity on day 7 of composting. Nevertheless, the rapid biodegradation of organic matter gradually deteriorated the anaerobic microenvironment within the compost pile. During the maturation stage, the nitrifying and denitrifying bacteria (*Pseudoxanthomonas* and *Bacillus*) were reactivated. Nitrification and denitrification gradually increased, leading to N_2_O production. Furthermore, a comparison with CK reveals that nitrate reduction of T2 was enhanced. This finding is consistent with the prediction depicted in Figure 4b, which suggests that the incorporation of Fe_3_O_4_ enhances the redox activity. Consequently, this enhancement facilitates the conversion of NO_3_^−^ to either NO_2_^−^ or N_2_. 

Network analysis revealed significant correlations between microorganisms and emissions of NH_3_ and N_2_O. In the CK group, *Romboutsia*, *Parabacteroides*, and *Prevotellaceae* exhibited positive correlations with NH_3_ emissions (Figure 6b). *Thermopolyspora* and *Methylococcaceae* were negatively correlated with NH_3_ emissions. Similarly, *Parabacteroides* and *Prevotellaceae* showed a negative association with N_2_O emissions. For the T1 treatment group, *Thermobifida* and *Chelativorans* displayed significant negative correlations with NH_3_ release. This finding is consistent with the observed increase in the abundance of *Thermobifida* and the reduction in ammonia emissions in the T1 group, suggesting that Fe_3_O_4_@BC influences NH_3_ emissions by modulating the microbial community. It was demonstrated that there was a negative correlation between *Ornithinicoccus* and *Cellvibrio*, and N_2_O emissions. The findings of this study indicate that the incorporation of Fe_3_O_4_@BC has led to alterations in key microorganisms, which in turn have had a subsequent influence on NH_3_ and N_2_O emissions.

### 3.7. Analysis of Mechanism

The ammonia-reducing effect of Fe_3_O_4_@BC during composting is likely mediated through its modulation of the microbial community structure, particularly by enriching functional taxa such as *Actinobacteria*. The addition of Fe_3_O_4_@BC did not significantly alter the physicochemical indicators of the composting system, including temperature, oxygen content and pH (Figure 2). Its function was mainly reflected in regulating the microbial community composition, thereby remarkably reducing nitrogen loss during the composting process. Compared with the control group, the addition of Fe_3_O_4_@BC significantly enriched *Actinobacteria*, which is closely associated with nitrogen cycling and extracellular electron transfer [43]. This is probably because Fe_3_O_4_@BC possesses strong electron donating capacity (EDC, Figure 1e), which promotes the increase in the abundance of *Actinobacteria*. A substantial increase was observed specifically in the abundance of the genus *Thermopolyspora* within *Actinobacteria* during the thermophilic phase in the T1 group. This shift towards *Actinobacteria* dominance under electrochemical stimulation aligns with findings by Zhong et al. [44], who reported that in electric field-coupled systems, *Actinobacteria*, alongside *Proteobacteria* and *Bacteroidetes*, can become co-dominant phyla, collectively comprising a large portion (e.g., ~80%) of the community. Concurrently, Fe_3_O_4_@BC addition appeared to suppress the activity of *Firmicutes*, potentially due to competitive inhibition by the prospering *Actinobacteria* [45]. Critically, the enrichment of *Actinobacteria* is functionally linked to ammonia reduction, as they can inhibit ammonifying bacteria via metabolic byproducts, thereby decreasing ammonia emissions [46]. This proposed microbial mechanism is consistent with the observed ammonia emission patterns (Figure 4a) and microbial network analysis (Figure 6) in this study. In essence, by serving as an effective electron conduit [45,47], Fe_3_O_4_@BC appears to stimulate the activity of key microorganisms like *Actinobacteria* while restraining others such as *Firmicutes*, thereby steering the compost microbiome towards a composition that favors nitrogen retention and minimizes gaseous loss.

Complementing its biological role, Fe_3_O_4_@BC exhibits a rough and porous surface with enhanced adsorption properties and abundant oxygen-containing functional groups (OCFGs), which are conducive to NH_3_ abatement. Previous studies have shown that the Fe_3_O_4_ modification increases material porosity, which not only optimizes the habitat for microbial growth but also confers high-efficiency NH_3_ adsorption capacity—a key physicochemical factor in emission reduction during composting. In summary, Fe_3_O_4_@BC facilitates green composting through two synergistic pathways: an Adsorption Pathway (porous structure & OCFGs → direct NH_3_ adsorption → emission reduction) and a Conductive Regulation Pathway (enhanced IET → microbial community modulation → emission reduction). The concurrent reduction in emissions of both NH_3_ and N_2_O across treatment groups corroborates the efficacy of this dual-mechanism strategy.

## 4. Conclusions

In this study, a porous Fe_3_O_4_@BC composite with a high specific surface area, abundant oxygen-containing functional groups, and excellent electron-donating capacity was successfully fabricated. The effects of the prepared material on nitrogen transformation and key microbial communities during electric field-assisted aerobic composting were systematically investigated [11]. This work provides novel functional materials and theoretical support for nitrogen retention, pollution reduction, and quality improvement in livestock manure composting.

Material characterization confirmed that Fe_3_O_4_ was uniformly loaded on the surface of biochar. The specific surface area of the composite was increased by 13.46% compared with pure biochar, accompanied by more developed microporous and mesoporous structures. New characteristic functional groups such as O–Fe–O were formed on the surface, and the EDC reached 4.8 times that of pure biochar. These structural and electrochemical properties endowed Fe_3_O_4_@BC with stronger NH_3_ adsorption capacity and higher electron donating capacity, laying a material foundation for regulating microbial nitrogen transformation and gas emission reduction. The results of nitrogen transformation and gas emissions demonstrated that the 1% addition group (T1) exhibited the optimal performance in emission reduction and nitrogen retention. Compared with the untreated group, the cumulative NH_3_ emission was reduced by 30.0%, total nitrogen loss was decreased by 4.03%, and N_2_O emission showed no significant increase, realizing synergistic emission control. Fe_3_O_4_@BC effectively inhibited the excessive accumulation of NH_4_^+^-N during the thermophilic phase, promoted the production of NO_3_^−^-N in the later stage, enhanced the stability and bioavailability of inorganic nitrogen, and drove the conversion of nitrogen from volatile forms to stable and utilizable forms. Microbiomic analysis revealed that under the coupling of electric field, Fe_3_O_4_@BC significantly reshaped the bacterial community structure: it enriched *Actinobacteriota* and restrained the proliferation of *Firmicutes* throughout the thermophilic period. At the genus level, the abundances of *Thermobifida* and *Corynebacterium* increased, whereas the relative abundance of ammoniation-dominant *Bacillus* decreased. The functional processes including nitrate reduction, nitrite ammonification, and nitrogen respiration were significantly enhanced in the treatment groups. Fe_3_O_4_@BC can directionally regulate key nitrogen-transforming microbiota and reduce gaseous nitrogen emissions at the metabolic level.

Overall, Fe_3_O_4_@BC achieved nitrogen retention and emission reduction in electric field-assisted composting through a dual-pathway synergy: first, physicochemical adsorption of NH_3_ due to its porous structure and abundant surface functional groups; second, based on the physicochemical properties of the material, nitrogen loss reduction is ultimately achieved through the regulation of microbial communities. This study confirms that Fe_3_O_4_@BC is a high-efficiency functional conditioner suitable for electric field composting. It provides a new technical solution for the resource utilization of biowaste, air pollution mitigation, and fertilizer quality improvement, and also offers theoretical basis and practical reference for the application of conductive composites in biological treatment systems.

## Figures and Tables

**Table 1:** Pore structure parameters of BC and Fe_3_O_4_@BC.

	BC	Fe_3_O_4_@BC
SSA (m^2^/g)	368.55	421.42
t-Plot micropore area (m^2^/g)	291.24	329.57
Total pore volume (cm^3^/g)	0.22	0.24
Average pore size (nm)	6.12	4.18

## Data Availability

All data generated or analyzed during this study are included in this published article. All data, models, and code generated or used during the study appear in the submitted article. The data that support the findings of this study are available from the corresponding author upon reasonable request.

## Supplementary Materials

The following supporting information can be downloaded at: https://www.mdpi.com/article/10.3390/nano16110689/s1, Table S1: Physicochemical properties of the raw materials; Table S2: Data of nitrogen loss path in composting.
