# Supplementary material for: Porous Fe3O4@BC Coupled with an Electric Field Facilitates Nitrogen Retention During Composting
Source: Nanomaterials (Basel). 2026 Jun 1;16(11):689. doi: 10.3390/nano16110689 (PMC13258605; doi:10.3390/nano16110689)
Supplement: Supplementary file 1 [file nanomaterials-16-00689-s001.zip › nanomaterials-4279669-supplementary.pdf]

# Supporting Information

## Microbial community analysis methods

Microbial community analysis was performed on samples collected on days 0, 3, 11, and 27. In accordance with the manufacturer's instructions, target DNA was extracted from the samples using a DNA extraction kit Omega Bio-tek, Norcross, GA, U.S), agarose gel electrophoresis, and a nucleic acid analyzer (NanoDrop2000, Thermo Scientific, United States). The key functional gene (16S rRNA V3–V4 region) of microbial communities was amplified with the primers 341F and 806R. The purified PCR products were subjected to library construction and sequencing using a sequence kit (nova-5149, NEXTFLEX) on the Illumina MiSeq PE300 platform (Majorbio, shanghai, China), and fastp software was used for data quality control. The processed sequences were clustered into operational taxonomic units (OTUs) via UPARSE software, followed by taxonomic annotation at a threshold of 70%. The microbial community composition of each sample was statistically analyzed at different taxonomic levels. PICRUSt2 software was used for the functional prediction of 16S rRNA gene sequences. Mothur software was applied to calculate the Alpha diversity indices, analyze intergroup differences, and conduct principal coordinate analysis (PCoA). Species for correlation network analysis were screened based on Spearman correlation coefficients.

The function of metabolically active bacteria were predicted by the FAPROTAX_1.2.1 database. The species abundance table derived from 16S rRNA sequencing was converted into a functional abundance table via standardized scripts. Strictly following the database matching rules, key functional groups involved in nitrogen transformation, including nitrification, ammoniation, etc, were screened. The relative abundances of nitrogen-transforming microorganisms were quantified through taxonomic homology mapping and abundance normalization, thereby clarifying the functional potential of environmental microorganisms in nitrogen cycling. All bioinformatic parameters were set uniformly to ensure a complete and reproducible analytical workflow, which guaranteed the scientificity and reliability of microbial sequencing and functional interpretation results.

## Calculation formula of nitrogen loss

The total nitrogen loss was calculated using the following formula:

$$\text{Nitrogen loss (\%)} = 100 - (X_2A_2) \times 100 / (X_1A_1) \qquad (1)$$

Where $X_1$ and $X_2$ represent the initial and final compost feedstock amount, and $A_1$ and $A_2$ denote the initial and final nitrogen content, respectively.

## Table S1 Physicochemical properties of the raw materials

| Sample | Moisture Content (%) | C content (%) | N content (%) | pH | EC (ms/cm) |
|---|---|---|---|---|---|
| **Pig mature** | 67.47 | 11.38 | 0.68 | 6.96 | 2.37 |
| **Corn straw** | 8.62 | 42.36 | 0.79 | - | - |

## Table S2 Data of nitrogen loss path in composting

| | T1 | | | T2 | | | CK | |
|---|---|---|---|---|---|---|---|---|
| **source** | **target** | **value** | **source** | **target** | **value** | **source** | **target** | **value** |
| TN | Material trans | **9.19%** | TN | Material trans | **11.37%** | TN | Material trans | **12.19%** |
| TN | $NH_4^+$ | **5.58%** | TN | $NH_4^+$ | **6.26%** | TN | $NH_4^+$ | **6.74%** |
| $NH_4^+$ | $NH_3$ | **3.83%** | $NH_4^+$ | $NH_3$ | **4.87%** | $NH_4^+$ | $NH_3$ | **5.49%** |
| $NH_4^+$ | $NO_3^-$ | **1.06%** | $NH_4^+$ | $NO_3^-$ | **0.96%** | $NH_4^+$ | $NO_3^-$ | **0.91%** |
| $NH_4^+$ | $N_2O$ | **0.02%** | $NH_4^+$ | $N_2O$ | **0.02%** | $NH_4^+$ | $N_2O$ | **0.02%** |
| $NH_4^+$ | Material trans | **0.67%** | $NH_4^+$ | Material trans | **0.41%** | $NH_4^+$ | Material trans | **0.32%** |
| $NO_3^-$ | Material trans | **1.06%** | $NO_3^-$ | Material trans | **0.96%** | $NO_3^-$ | Material trans | **0.91%** |
| Material trans | Other loss | **9.19%** | Material trans | Other loss | **11.37%** | Material trans | Other loss | **12.19%** |
| Material trans | Inorganic-N | **1.73%** | Material trans | Inorganic-N | **1.39%** | Material trans | Inorganic-N | **1.24%** |
| $NH_3$ | TN loss | **3.83%** | $NH_3$ | TN loss | **4.87%** | $NH_3$ | TN loss | **5.49%** |
| $N_2O$ | TN loss | **0.02%** | $N_2O$ | TN loss | **0.02%** | $N_2O$ | TN loss | **0.02%** |
| Other loss | TN loss | **9.19%** | Other loss | TN loss | **11.37%** | Other loss | TN loss | **12.19%** |
